# Usefulness of PKH fluorescent labelling to study leukemic cell proliferation with various cytostatic drugs or acetyl tetrapeptide – AcSDKP

**DOI:** 10.1186/1471-2407-5-120

**Published:** 2005-09-20

**Authors:** Jean Boutonnat, Anne-Marie Faussat, Jean-Pierre Marie, Jérôme Bignon, Johanna Wdzieczak-Bakala, Magali Barbier, Josiane Thierry, Xavier Ronot, Pierre-Emmanuel Colle

**Affiliations:** 1 Laboratoire de Dynamique Cellulaire, E.P.H.E, IFRT 130, CNRS UMR 5525, 38706 La Tronche cedex, France; 2 Département d'Hématologie-Oncologie, Hôtel Dieu, 75004 Paris, France; 3 Institut de Chimie des Substances Naturelles, CNRS, avenue de la Terrasse, 91198, Gif-sur-Yvette, France; 4 Département de Langue, Université Joseph Fourier, 38706 La Tronche cedex, France

## Abstract

**Background:**

PKH67 labelling was compared for classical proliferation assessment (using S phase evaluation) to analyse the cell proliferation of 29 AML patients treated or not with various drugs. Among these drugs, the effect of tetrapeptide AcSDKP or AcSDKP-NH2 on AML cells, stimulated or not by cytokines, was also evaluated in order to determine (i) if AcSDKP was able to inhibit blast cell proliferation as it inhibits haematopoietic progenitors (ii) if AcSDKP-NH2 was more stable than AcSDKP with FBS.

**Methods:**

For PKH labeling, cells were suspended in Diluent C, and rapidly admixed with PKH67 solution at 20 μM PKH67. Staining was stopped by addition of FBS.

**Results:**

A good correlation between PKH67 labelling and bromodeoxyuridine incorporation was obtained first with 6/9 patients for control cells, then for 11/17 AML patients treated with classical antileukemic drugs (among whom 4 were also treated with AcSDKP). The effect of AcSDKP was also studied on 7 patients. The discrepancy between both methods was essentially due to an accumulation of cells into different cycle phases measured by BrdUrd incorporation secondary to drug action and PKH67 labelling which measured the dynamic proliferation. This last method allows identifying resistant cells which still proliferate. AcSDKP or AcSDKP-NH2 induced a decrease of leukemic cell proliferation in 5/7 patients when cytokines were added (in order to stimulate proliferation) one day after tetrapeptide AcSDKP or AcSDKP-NH2. No effect on proliferation was noted when cytokines were added to AcSDKP-NH2.

**Conclusion:**

PKH67 labelling method is a powerful tool for cell proliferation assessment in patients with AML, even in cells treated by various drugs.

## Background

The successful treatment of acute myeloid leukaemia (AML) is frequently impeded by the development of resistance to a wide spectrum of cytotoxic drugs and by cell proliferation. Daunorubicin (DNR), Cytarabine (AraC), Etoposide (VP16), Mitoxantrone (Mitox), and Amsacrin (AMSA) are used in the treatment of AML and can induce drug resistance [1]. Various methods are available to assess leukemic cell proliferation. Common methods for proliferation assessment, such as bromodeoxyuridine (BrdUrd) incorporation, are correlated to treatment outcome [2,3]. BrdUrd is an analogue of thymidine and can be incorporated specifically into DNA instead of thymidine. BrdUrd incorporation was described, in literature, as a reference technique for cell proliferation evaluation but is often difficult to standardize [3]. Evaluation of cell distribution in each phase could also be determined by monoparameter analysis after stoichiometric DNA labelling using propidium iodide (PI) [4]. These two methods require cell fixation and cell permeabilization whereas PKH dye labelling can be performed on living cells.

PKH (from the author who developped these dyes: Paul Karl Horan) are vital lipophilic, fluorescent, membrane intercalating dyes [5]. They contain two long alkyl chains, which allow a strong anchorage in the lipid bilayer. When labelled cells divide, the resulting daughter cells receive half the label, reducing the fluorescence intensity to one-half that the parent cells. As a consequence, the proliferation of labelled cells is correlated to a decrease in fluorescence [6,7]. Drugs such as DNR, a fluorescent molecule, do not interfere with PKH67 staining, when a delay (3 hours minimum) between PKH67 labelling and DNR incubation is respected [8].

The tetrapeptide acetyl-N-Ser-Asp-Lys-Pro (AcSDKP) isolated from bone marrow was identified as a physiological regulator of hematopoietic stem cell proliferation [9]. It inhibits the proliferation of normal haematopoietic stem cells and early progenitors *in vivo *as well as *in vitro *[10-12]. However, the AcSDKP role on cell proliferation has been discussed. Some authors have reported that AcSDKP has no effect on the proliferative status of leukemic progenitors [11] and therefore may selectively prevent the cycle initiation of normal stem cells.

Recent studies have reported that AcSDKP is inactivated by foetal bovine serum (FBS). It is hydrolyzed in blood by the soluble angiotensin-I converting enzyme (ACE) [13]. A new AcSDKP (AcSDKP-NH2) was developed to increase its stability against ACE degradation in FBS and blood. Therefore, it was interesting to know if this analogue also shared common properties with AcSDKP on the proliferation status of leukemic cells.

The aim of this study was to compare the proliferation of 29 AML cells from patients treated or not with cytostatic drugs using two methods: i) dye dilution method using PKH67 ii) or DNA content. The AcSDKP or AcSDKP-NH2 effect on cell proliferation was analyzed.

## Methods

### Reagents

Ficoll, PKH67 and Diluent C were given by Sigma-Aldrich (St Quentin Fallavier, France). Daunorubicin (DNR), Aracytine (AraC) and Amsacrine (AMSA) were purchased from Roger-Bellon (Neuilly sur Seine, France). Mitoxantrone (Mitox) was given from Léderlé (Rungis, France). VP16 was supplied by Pierre-Fabre (Castres, France). BrdUrd and anti-BrdUrd were purchased from Roche-Diagnostic (Meylan, France). Cell growth medium, fetal bovine serum (FBS), phosphate-buffered saline (PBS), and cellulose syringe filters (0.45 mm) were purchased from Invitrogen (Cergy-Pontoise, France).

Recombinant granulocyte-macrophage colony stimulating factor (GM-CSF) and granulocyte colony stimulating factor (G-CSF) were used at a concentration of 20 ng/mL. Interleukin 3 (IL3) was used at 50 ng/mL. The stem cell factor (SCF) concentration was 25 ng/mL, and recombinant human ertyhropoietin (EPO) was used at 1.5 UI/mL. All growth factors were purchased from Biosource (California, USA).

The synthetic tetrapeptide (Acetyl-N-Ser-Asp-Lys-Pro) or AcSDKP-NH2 was generously donated by J. Bignon and J.Thierry respectively (Institut de Chimie des Substances Naturelles, CNRS, France).

### AML cells sampled from patients

Leukemic cells were obtained from peripheral blood or bone marrow samples in 29 AML patients, separated on a gradient of density (Ficoll). Only the patients samples with more than 50% of living blasts were included in the experiments. The 29 patients were studied as follow: 9 using BrudUrd incorporation and PKH67 labelling without drug treatment, 17 using BrudUrd incorporation and PKH67 labelling in the presence of drug or not; among the 18 patients, 4 were studied along with the remaining 3 using PKH67 labelling and DNA content analysis in order to determine AcSDKP or AcSDKP-NH2 effect.

Cells were cultured in RPMI-1640 medium supplemented with 10% FBS, 2 mM glutamine, 100 UI/mL penicillin, and 100 ng/mL streptomycin, at 37°C in a humidified atmosphere with 5% CO2.

Analysis were performed every day, as long as the percentage of viable cells was higher or equal to 50%. Viable and necrotic cells were identified, using flow cytometry with forward and side scatter parameters.

### PKH67 labelling

PKH67 labelling was performed as described previously [14]. Briefly, 10^7 ^cells were suspended in 1.0 mL of Diluent C, and stained by rapidly admixing with a 20 μM working PKH67 solution, prepared by diluting 20 μl of 10^-3 ^M ethanolic dye stock in 1.0 mL of Diluent C immediately prior to staining. Final staining concentration was therefore 10 μM PKH67 and 5 × 10^6 ^cells/mL). Staining was stopped after 3 minutes by addition of 2 mL of FBS and cells were washed 3 times with 5 mL of RPMI-1640 containing 10% FBS. For each sample, an aliquot of blasts was fixed with 2% PFA at D0 and kept at +4°C in order to maintain the original fluorescence.

### BrdUrd incorporation

10^7 ^cells were cultured in complete medium for each sample. After 24 hours, 5 μM BrdUrd were incorporated in control cells for 15 minutes at 37°C. After elimination of excess BrdUrd by 2 washings, cells were fixed by 70° ethanol and maintained at +4°C during 24 hours. Revelation of BrdUrd molecules was made by treating cells with hydrochloric acid 4N during 15 minutes at room temperature. After several washings, 20 μL of Ac anti-BrdUrd [15] were added to control cells for 30 minutes at room temperature. Ac anti-BrdUrd was revealed by Fab'_2 _(antibody coupled with FITC, 0.4 μg/mL) for 30 minutes at room temperature. Cells were labeled simultaneously with 10 μg/mL PI and treated with RNase (1 mg/mL) during 30 minutes at room temperature, then analyzed using a FACSCalibur (BD Biosciences, le Pont de Claix, France) to measure DNA content (G0/1 and G2+M phases).

### Evaluation of blast proliferation following drug treatment

Blasts were collected and labeled with PKH67. Cells were treated during four days with one or the combination of several drugs after one day of culture: DNR, AraC, AMSA, VP16, and Mitox, at a concentration of 10^-6 ^M. These concentrations are commonly used in therapy.

### AcSDKP effect on proliferation of cells (stimulated or not by cytokines)

2.10^6 ^labelled with PKH67 were seeded in 25 cm^2 ^culture dishes numbered as described below:

Sample 1: PKH67 labelled cells without the mixture of cytokines in complete medium at 37°C.

Sample 2: PKH67 labelled cells were cultured with cytokines in complete medium at 37°C (G-CSF, IL3, SCF, GM-CSF, and EPO).

Sample 3: cells with cytokines and 10^-9 ^M AcSDKP-NH2 in complete medium at 37°C.

Sample 4: cells without cytokines and 10^-5 ^M AcSDKP in complete medium at 37°C.

Sample 5: cells without cytokines and 10^-9 ^M AcSDKP in complete medium at 37°C.

Sample 6: cells without cytokines and 10^-9 ^M AcSDKP-NH2 in complete medium at 37°C.

Sample 7: cells without cytokines and 10^-9 ^M AcSDKP-NH2 in complete medium at 37°C. After a day of culture, cells contained in samples 4, 5, and 6 were washed and cytokines were added to the complete medium.

The tetrapetide AcSDKP concentrations used upper are known to be active.

### DNA content analysis

The cell cycle was evaluated with the Cycle Test™ kit (BD-Biociences, Le Pont de Claix, France). Briefly, cells from patients were incubated with trypsin in a spermine tetrahydrochloride detergent buffer for 10 min at room temperature. Trypsin inhibitor and ribonuclease A were added for 10 min without washing. Finally, PI was added and incubated for 10 min, then cells were immediately analyzed by flow cytometry and distribution of cells in each phase was evaqluated using Modfit software (Verity software). Since both tetrapeptide could induce an increase of cells in G0/1 phase with the Cycle Test™ kit was used because it was easier than Brdu incorporation to evaluate few modification in cell cycle repartition.

### Flow cytometry analysis

Mean fluorescence intensity per cell was measured using a FACSCalibur flow cytometer (BD Biosciences, Le Pont de Claix, France) equipped with an air-cooled argon ion laser emitting 15 mW at 488 nm and a photodiode laser emitting 10 mW at 635 nm. PKH67 and FITC fluorescence were collected with a 530 ± 30 nm band-pass filter; PI fluorescence was collected with a 585 ± 44 nm band-pass filter. 50,000 events were acquired with the CellQuest software (BD Biosciences, Le Pont de Claix, France). Electronic compensation settings for FITC and PI were FL1-FL2 = 10% and FL2-FL1 = 35%; and FL1-FL2 = 8% and FL2-FL1 = 30% for PKH67/PI. Kolmogorov Sminov statistical test (K/S test) was used to point out difference between PKH fluorescence histograms. Differences between histograms were considered as statistical significant when p was under 0.001.

## Results

**Figure 1:**
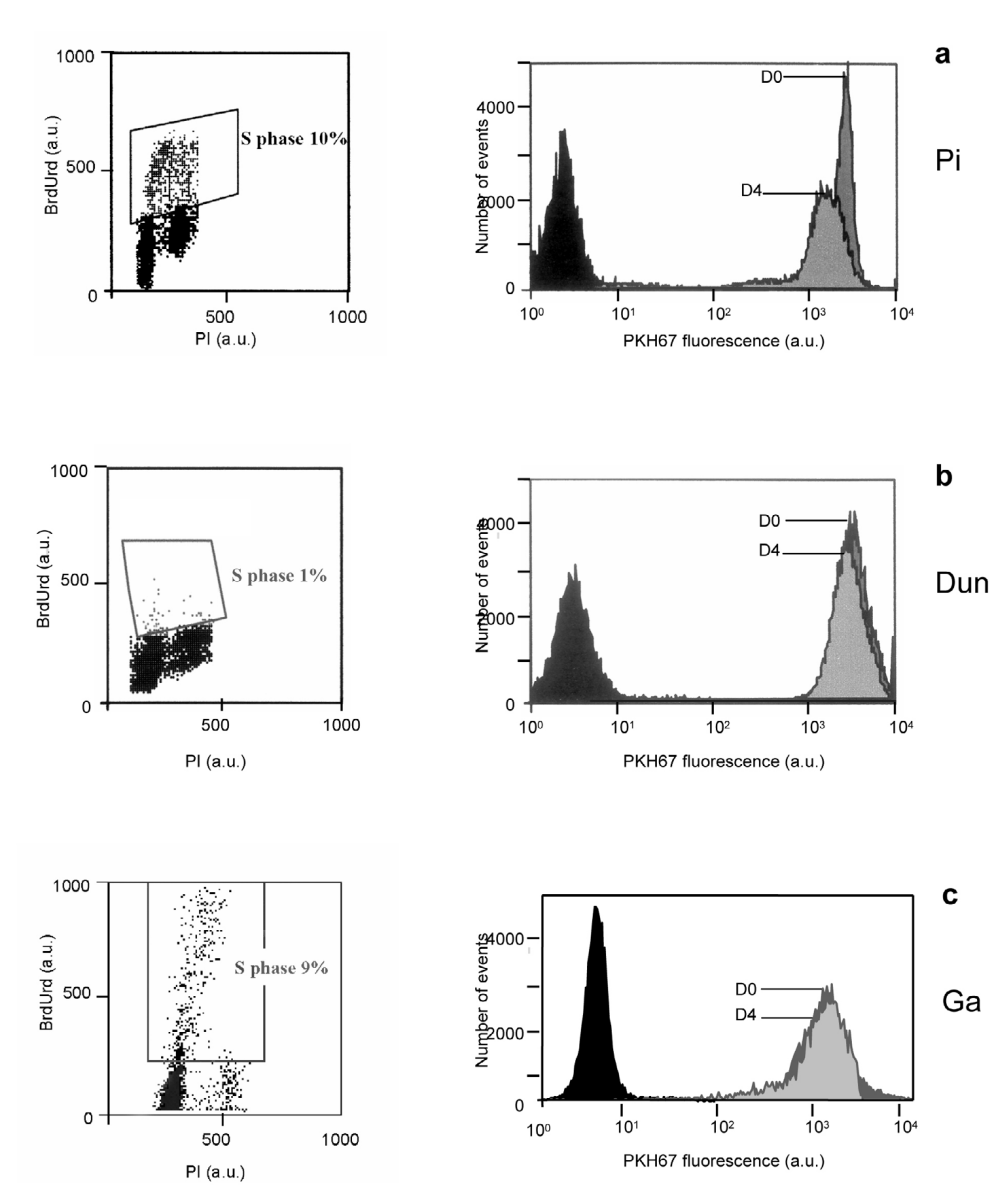
Comparative flow cytometry analyses of percentage of cells in S phase, using BrdUrd incorporation and PKH67 labelling {PKH fluorescence intensity (a.u.) at Day 0 / PKH fluorescence intensity (a.u.) at Day 4}. Figure 1a patient Pi, Figure 1b patient Dun, Figure 1c patient Ga.

We compared blast proliferation with two methods: a reference method (BrdUrd incorporation) and a dye dilution method (PKH67 labelling), in order to validate the use of PKH for proliferation assessment of living AML cells. Table 1 shows the cell cycle distribution and the mean PKH67 fluorescence of cells from 9 patients. Fluorescence ratio is, PKH67 fluorescence at D0 divided by PKH67 fluorescence at D4 (D0/D4). Three different groups could be described according to the proliferation rate determined by PKH67 decrease and BrdUrd incorporation i) first group with proliferation corresponding to PKH67 decrease and an S phase superior to 4% ii) second group with low S phase (1%) and PKH67 ratio under 1.2 and iii) a third group with a discordance between S phase and PKH67 decrease. Using the Kolmogorov Sminov test we showed that with a PKH67 fluorescence ratio at 1.21 there was a significant difference between histograms [p < 0.001 for patients (Lio., Pi., Tr., Gr.)] but not below this value. We considered that cells proliferated when PKH67 fluorescence ratio was superior to 1.21. In the first group of patients, (Lio., Pi., Tr., Gr.) PKH67 fluorescence ratios were 2.96, 1.37, 1.39, 1.29 respectively (table 1) and showed a cell proliferation correlated to the S phase fraction (30% to 4%) (Figure 1a).

In the second group, patients Dun and Br (Figure 1b) showed a low decrease of PKH67 fluorescence, with a fluorescence ratio (1.15 and 1.16) respectively at D0/D4. The cell cycle analysis showed that 1% of the cells was in S phase (Figure 1b).

In the third group, the S phase ranged from 8 to 11% but the PKH67 ratio ranged from 1.06 to 1.16. These 3 patients showed a discrepancy between PKH67 fluorescence, revealing an interruption of proliferation and S phase (Figure 1c).

### Evaluation of blast proliferation treated with various drugs (Table 2)

In this part of experimentation, 17 patients were analysed. According to the initial results shown in Table 1 and the K/S test, three groups were identified: (i) cells from 4 patients which still underwent proliferation even after drug treatment (ii) cells from 6 patients which were more or less sensitive to one or more drugs associated with interruption of proliferation, and (iii) 7 patients with no spontaneous proliferation.

**Figure 2:**
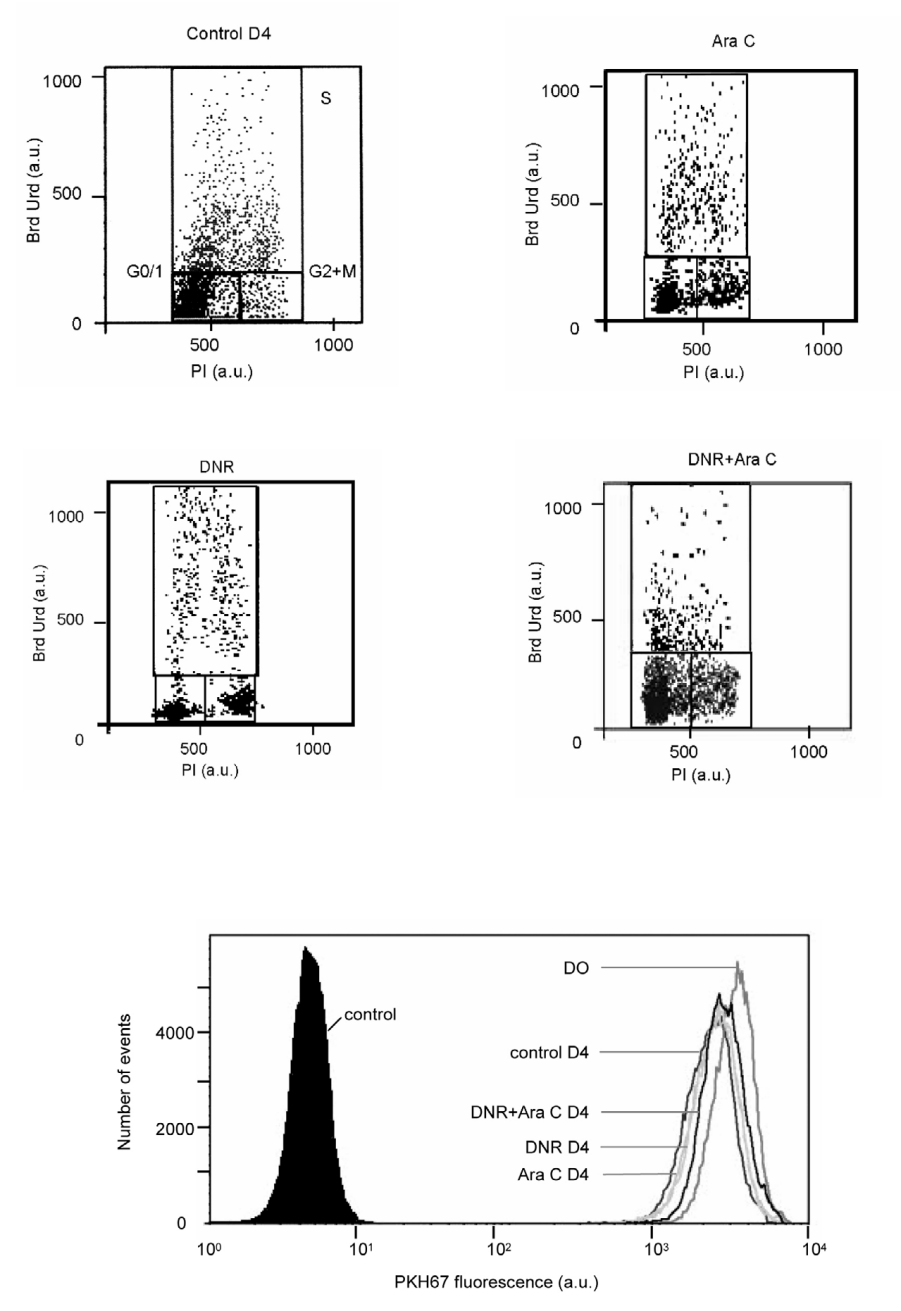
Comparative flow cytometry analyses of percentage of cells in S phase using BrdUrd incorporation and PKH67 ratio in patients Gi., treated with DNR, AraC, and a mixture of DNR+AraC after 4 days.

Cells from patient Gi presented a moderate spontaneous proliferation (PKH67 ratio at 1.4 and 8% of cells in S phase) (Figure 2). When cells were treated with AraC, DNR, or both no decrease of PKH67 ratio was noted. Small modifications of cell cycle phase distribution were seen essentially with DNR or DNR + AraC with an accumulation in G2+M phase.

For the cells of patient Lh, a decrease of the PKH67 ratio compared to the control (3.7) was noted for AraC (2.32), DNR (2.09), or combination of both (1.92) but cells still underwent proliferation. When cells were treated with AraC a moderate accumulation was seen in the S phase (39%) and an accumulation in G2+M phase for DNR (30%) or DNR+ AraC (24%). For both patients, Da and Go, no accumulation was noted in one of the cell cycle phases but with AraC a moderate decrease of proliferation kinetics was pointed out by a decrease of PKH67 fluorescence ratio (1.4 and 1.3 respectively) compared to the control (1.6).

**Figure 3:**
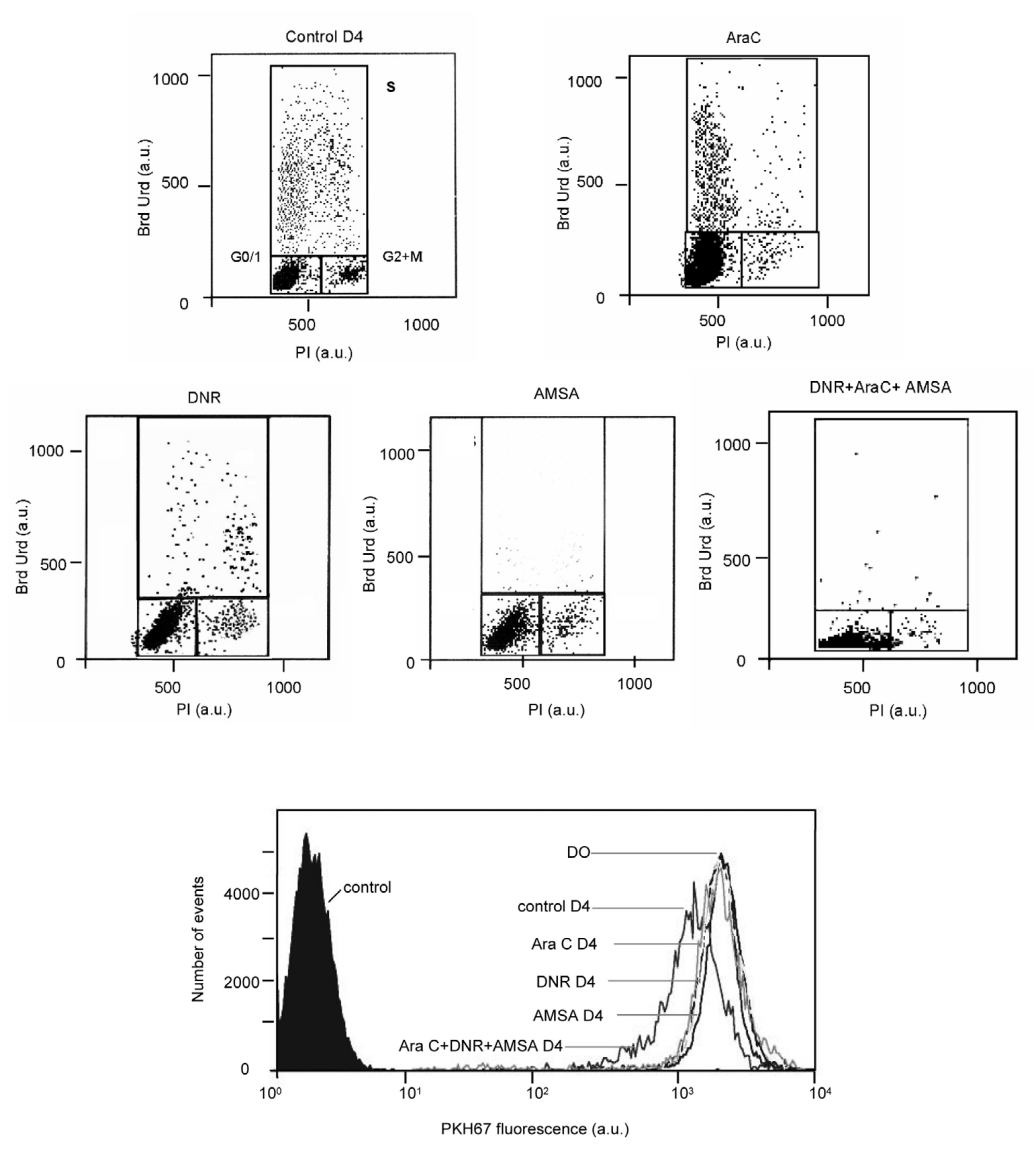
Comparative flow cytometry analyses of percentage of cells in S phase using BrdUrd incorporation and decrease of PKH67 fluorescence ratio in patient Fo., treated with DNR, AraC, AMSA, and a mixture of DNR+AMSA+AraC after 4 days.

In the second group Sat, Le, Fo (figure 3), and Ko cells had spontaneous proliferation but each drug interrupted proliferation. More often AraC induces an accumulation in G1 or S phase and the others drugs, Amsacrine, daunorubicine, or VP16 induced an accumulation in G2+M. When drugs were combined, accumulation in one cell cycle phase was not obvious. For patients Sa and Na, treatment with AraC induced a decrease of proliferation shown by a PKH67 fluorescence ratio decrease, 1.4 and 2.5 respectively compared to 3 for the control. As for other patients in the group, DNR interrupted the proliferation with a PKH ratio under 1.2. In the third group no spontaneous proliferation was noted "a fortiori" under treatment.

### The evaluation of AcSDKP on blast proliferation stimulated or not by cytokines (Table 3)

We used the PKH67 assay and Cycle test from BD to assess proliferation on blasts incubated with cytokines, AcSDKP or AcSDKP-NH2.

In this experiment, two groups of patients could be individualized: three patients underwent spontaneous proliferation (Be, Dur, Ko), and the four others (Pa, Ph, Al, Av) underwent proliferation only after cytokine stimulation.

The proliferation of cells from patient Be was not more stimulated by adding cytokines compared to the control and no effect of AcSDKP or AcSDKP-NH2 was noted on PKH67 fluorescence ratio or on the cell cycle phase. Cells obtained from Dur patient were stimulated by cytokines and an increase of proliferation was noted according to the increase of PKH67 fluorescence ratio (1.5 compared to the control 1.2) and no effects of AcSDKP or AcSDKP-NH2 was noted on cell cycle phase or PKH67 fluorescence ratio. Cells from patient Ko were stimulated by cytokines but if AcSDKP or AcSDKP-NH2 was added a day before cytokines (sample 3) cell proliferation was interrupted along with a decrease of the S phase associated with an increase of G0/1 phase and a decrease of PKH67 fluorescence ratio (1 for sample 4, 1.12 for sample 5, 1.11 for sample 6 compared to the control 1.49 sample 2).

**Figure 4:**
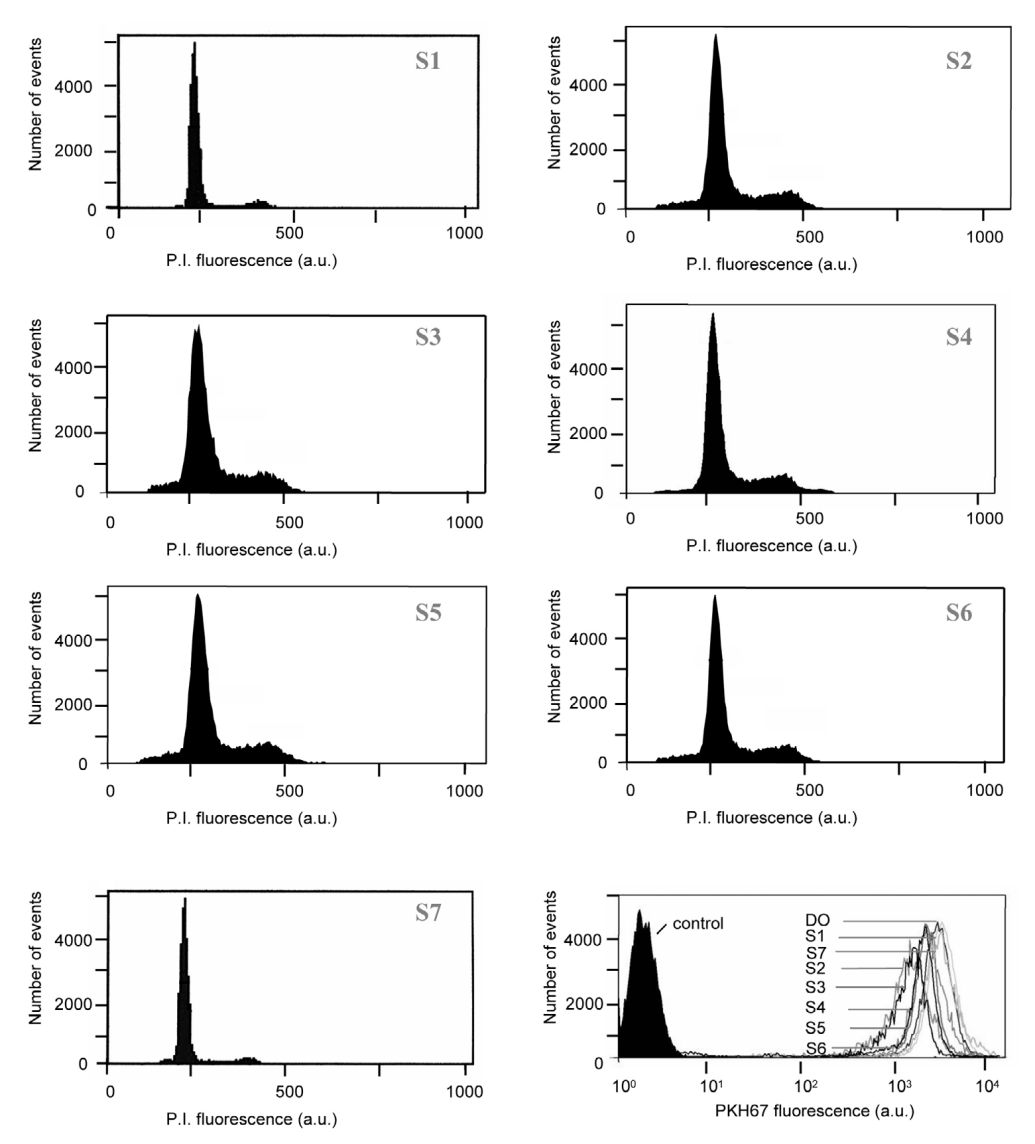
Comparative flow cytometry analyses using PKH67 fluorescence ratio (PKH^®^) and cell cycle of patient Ph. treated with cytokines and AcSDKP after 4 days of culture.

In the second group, cells from patients Pa and Ph (Figure 4) underwent proliferation after cytokine stimulation but if AcSDKP or AcSDKP-NH2 was added a day before adding cytokines a slow decrease of proliferation was noted (0.2 in the fluorescence ratio in sample 4,5,6 compared to the ratio of sample 2 or 3). No obvious difference was noted in the proportion of S phase in the various samples. For the two last patients (Al and Av) AcSDKP or AcSDKP-NH2 which was added one day before adding cytokines interrupted the proliferation with a PKH67 fluorescence ratio and a percentage of S phase close to 1.

## Discussion

This study had for aim to compare cell proliferation of 29 AML patients by two methods i) S phase evaluation and DNA content for proliferation assessment and ii) PKH67 labelling to monitor proliferation of living blast treated or not cytostatic drugs or AcSDKP a regulator of stem cells.

The proliferation assessment, using BrdUrd incorporation and PKH67 labelling, was carried out on 9 AML patients, 7 of whom had been recently diagnosed and the other 2 were relapsing. In order to compare results, the K/S test was applied to point out statistical difference between PKH67 fluorescence histograms. Results were significant when the ratio of the PKH67 histograms fluorescence was above 1.2. In the first group of patients, the proliferation was highly significant identified by BrdUrd incorporation and by a decrease of PKH67 fluorescence. These results show a good correlation between the S phase percentage and proliferation rate. In the second group, no objective proliferation was shown either with PKH67 labelling or S phase. In the third group a discrepancy was noted between the S phase evaluation and proliferation using PKH67 labelling with high S phase and no significant proliferation with PKH67 labelling, The discrepancy between the two methods can be explained by the fact that BrdUrd incorporation associated to the measure of the DNA content, allows to estimate cell distribution in various phases like a snapshot, as opposed to PKH67 which measures cell division. The evaluation of dynamic proliferation, using BrdUrd incorporation, would require using the "pulse – chase" method which is time-consuming and difficult to set-up [16].

The correlation between PKH67 labelling and the rate of S phase cells had to be validated when cells are cultured with cytostatic drugs. Indeed out of 17 AML patients, treated with drugs, 11 showed a good correlation between the S phase percentage of cells and the decrease of PKH67 fluorescence. In the first group of patients (4 cases), a significant proliferation of cells was observed when cells were treated with various drugs. This proliferation of cells treated by DNR, VP16, Mitox could be due to ATP binding cassette proteins such as Pgp, MRP, and BCRP [17-19]. Pgp, MRP, BCRP are known to extrude DNR, VP16, and Mitox. The proliferation of cells treated by AraC could be explained by spliced deoxycytidine kinase [20]. F. Lacombe *et al*., [2], reported that a minimum of 3% of cells in phase S was necessary to show a significant difference, in a study of DNA synthesis inhibition of by AraC. These results are correlated to our results obtained with the K/S test. AraC, a drug often used in therapy [21], was described in literature as an antimetabolite involving the S phase accumulation of cells [22,23].

In the second group, discrepancies were seen between the percentage of S phase and the PKH67 fluorescence ratio in the presence of AraC, in two cases. The S phase percentage was superior to 20% with a BrdUrd/FITC and PI biparametric analysis. The PKH67 fluorescence histogram did not reveal any cellular proliferation. In these cases, cells seemed to accumulate in phase S. The same profile was observed when cells were treated with the DNR+AraC mixture. Both drugs were in competition and only AraC dominated, because an accumulation of cells was obtained in phase S and not in phase G2+M. We did not observe cumulative effects between these two drugs. VP16 and AMSA are inhibitors of topo-isomerases II and we observed a low decrease of PKH67 fluorescence in 11 cases, thus a low rate of proliferation correlated to the S phase percentage of cells. We did not observe any modification of cell cycle distribution in the presence of these drugs. These two drugs were reported as inducing cell accumulation in G2+M [24,25].

We thus studied a possible effect of the tetrapeptide AcSDKP or the amide AcSDKP (which was supposed to be more stable) on proliferation of AML blasts, previously stimulated or not by cytokines. AcSDKP isolated from bone marrow was identified as a physiological regulator of cell proliferation [9]. AcSDKP is known to inhibit cell proliferation of normal haematopoietic *in vivo *and *in vitro *[10,26]. However, it was described as being ineffective on the proliferation of leukemic cells [27] and thus it could selectively prevent the cell cycle initiation. It was also described as a protector of normal haematopoietic human cells, against the toxic effects of drugs, and the effects of radiotherapy [28-31]. It has no effect on the growth and DNA synthesis of HL60 leukemic cells [27]. The biological properties of AcSDKP and the absence of antiproliferative activity on leukemic cells suggest that possible therapeutic applications, such as the protection of hematopoietic cells, could be used in association with chemotherapy. The Institute of Chemistry of Natural Substances (CNRS, Gif-sur-Yvette, France.) developed a tetrapeptide amide to limit degradation by an ACE enzyme when cells are cultured with FCS or blood [32]. We demonstrated that there was no difference between the tetrapeptide amide and the natural tetrapeptide. This could be explained by the fact that the medium used for the culture of blastic cells contained only 10% of FCS. AcSDKP had no effect on the proliferation of stimulated cells in 5 patients [27], as well as on leukemic HL60 and K562 cells lines, and on CML cells [31,32]. We observed a low rate of, or an inhibition of proliferation in 3 patients when cytokines were added one day after AcSDKP. M Smeets *et al*. [33] reported that "noncycling" progenitors, whether normal or leukemic, presented a relatively increased level of MDR protein expression. We probably modified the expression of resistant proteins when we activated the proliferation of these cells, by a decrease of resistant protein expression. P Te Boekhorst *et al*., [34] showed the existence of a relationship between the Pgp protein and a high fraction of cells in phase S. They showed that high S phase was frequently associated with the expression of multidrug resistance proteins and poor prognosis, in acute myeloid leukaemia.

## Conclusion

Our study more frequently demonstrated a good correlation between PKH 67 fluorescence decrease and proportion of S phase obtained by BrdUrd incorporation. We observed a discrepancy when cells were blocked in one of the cell cycle phases and demonstrated the power of PKH67 labelling to follow proliferation even when cells were treated with drugs. This method could be applied to sort proliferating cells growing with drugs and to determine their chemoresistant protein profiles. We also demonstrated the absence of benefit of the NH2 AcSDKP compared to AcSDKP since both are able to modify blast cell proliferation which prevents using them in therapy.

## Abreviations

PKH: Paul Karl Horan, BrdUrd: bromodeoxyuridine, PI: propidium iodide, AcSDKP: tetrapeptide acetyl-N-Ser-Asp-Lys-Pro, NH2AcSDKP : tetrapeptide acetyl-N-Ser-Asp-Lys-Pro amide, DNR: Daunorubicin, AraC: Aracytine, AMSA: Amsacrine, Mitox: Mitoxantrone, VP16: Etoposide.

## Competing interests

The author(s) declare that they have no competing interest.

## Authors' contributions

A-M F acquired and analyzed part of the data. J Bi, and J W provided drugs. M B wrote the first draft of the manuscript. J Bo, J-P M, and X R conceived and the designed the study and provided guidance to all aspects of this project. English revision of the maniuscript was done by P-E C. All authors read and approved the final manuscript.

## Pre-publication history

The pre-publication history for this paper can be accessed here:



## Figures and Tables

**Table 1:** Comparison between BrdUrd incorporation asociated with DNA content (% of cell in cell cycle) at day 4 and PKH67 labelling ratio {PKH fluorescence intensity (a.u.) at Day 0 by PKH fluorescence intensity at Day 4} in 9 AML patients.

**Patients**	**Cell cycle**			**Cell proliferation**		
	**G0/1%**	**S%**	**G2+M %**	**D0 (a.u.)**	**D4 (a.u.)**	**PKH ratio**
***Lio***	47	30	23	579	194	2.96
***Pi***	72	10	18	1966	1424	1.37
***Tr***	66	11	23	1851	1324	1.39
***Gr***	76	4	20	1988	1324	1.21
***Dun***	64	1	35	4821	4138	1.16
***Br Relapse***	76	1	23	3960	3438	1.15
***Li***	69	11	20	3020	2834	1.07
***Ga***	81	9	10	1995	1853	1.06
***To***	69	8	23	1444	1257	1.16

**Table 2:** Comparison between BrdUrd incorporation and PKH67 labelling in 17 AML patients treated with various drugs (DNR, AraC, VP16, Mitox, and AMSA). Percentage distribution of patient cells in each cell cycle phase obtained by BrdUrd incorporation and PKH67 labelling ratio at day 4 (PKH fluorescence intensity at Day 0 by PKH fluorescence intensity at Day 4).

***GI***	**Control**	**AraC**	**DNR**	**AraC + DNR**	
**G0/1**	89	88	74	72	
**S**	8	6	8	4	
**G2+M**	3	6	18	24	
**PKH ratio**	1,4	1,35	1,37	1,25	
***Lh***	**Control**	**AraC**	**DNR**	**AraC + DNR**	
**G0/1**	57	53	54	53	
**S**	33	39	16	23	
**G2+M**	10	8	30	24	
**PKH ratio**	3,7	2,32	2,09	1,93	
***Da***	**Control**	**AraC**	**VP16**	**Mitox**	**AraC + VP16 + Mitox**
**G0/1**	74	76	66	73	71
**S**	7	10	10	12	17
**G2+M**	19	14	24	15	12
**PKH ratio**	1,6	1,4	1,4	1,5	1,4
***Go***	**Control**	**AraC**	**VP16**	**Mitox**	**AraC + VP16 + Mitox**
**G0/1**	60	64	85	71	75
**S**	16	10	4	8	2
**G2+M**	24	26	11	21	23
**PKH ratio**	1,6	1,3	1,6	1,6	1,3
***Sat***	**Control**	**AraC**	**VP16**	**Mitox**	**AraC + VP16 + Mitox**
**G0/1**	82	87	89	89	89
**S**	14	7	4	1	1
**G2+M**	4	6	7	10	10
**PKH ratio**	1,7	1,1	1,1	1,1	1,1
***Le***	**Control**	**AraC**	**VP16**	**Mitox**	**AraC + VP16 + Mitox**
**G0/1**	70	61	56	77	77
**S**	14	19	16	2	1
**G2+M**	16	20	28	21	22
**PKH ratio**	2,3	1,12	1,07	1,05	1,05
***Fo***	**Control**	**AraC**	**AMSA**	**DNR**	**DNR+AraC+AMSA**
**G0/1**	81	75	90	71	90
**S**	14	20	2	6	2
**G2+M**	5	5	8	23	8
**PKH ratio**	1,7	1,1	1,18	1,18	1,18
***Ko***	**Control**	**AraC**	**DNR**	**VP16**	**AraC+DNR+VP16**
**G0/1**	71	80	70	74	76
**S**	13	2	8	4	3
**G2/M**	16	18	22	22	21
**PKH ratio**	1,5	1,05	1,13	1,04	1,06
***Sa***	**Control**	**AraC**	**DNR**	**AraC+DNR**	
**G0/1**	56	71	76	76	
**S**	32	10	9	5	
**G2+M**	12	19	17	19	
**PKH ratio**	3	1,4	1,1	1,12	
**Na**	**Control**	**AraC**	**DNR**	**AraC+DNR**	
**G0/1**	65	74	57	73	
**S**	31	21	1	23	
**G2+M**	4	5	42	4	
**PKH ratio**	3	2,5	1,13	1,11	
***Pa***	**Control**	**AraC**	**DNR**	**AraC+DNR**	
**G0/1**	89	90	87	89	
**S**	3	1	1	1	
**G2+M**	8	9	12	10	
**PKH ratio**	1,18	1,1	1,1	1,1	
***La***	**Control**	**AraC**	**DNR**	**AraC+DNR**	
**G0/1**	83	85	60	80	
**S**	11	7	3	13	
**G2+M**	6	8	37	7	
**PKH ratio**	1,02	1,01	1,1	1,1	
***Luu***	**Control**	**AraC**	**DNR**	**AraC+DNR**	
**G0/1**	86	82	86	85	
**S**	1	1	2	3	
**G2+M**	13	17	12	12	
**PKH ratio**	1,1	1,12	1,13	1,12	
***Av***	**Control**	**AraC**	**DNR**	**AraC+DNR**	
**G0/1**	68	65	80	68	
**S**	20	16	13	15	
**G2+M**	12	19	7	17	
**PKH ratio**	1,09	1,07	1,08	1,1	
***Ph***	**Control**	**AraC**	**DNR**	**AraC+DNR**	
**G0/1**	74	75	77	79	
**S**	1	2	3	1	
**G2+M**	25	23	20	20	
**PKH ratio**	1,1	1,03	1,05	1,07	
***Li***	**Control**	**DNR**			
**G0/1**	70	73			
**S**	11	7			
**G2+M**	19	20			
**PKH ratio**	1,07	1,08			
***To***	**Control**	**DNR**			
**G0/1**	92	73			
**S**	5	5			
**G2+M**	3	22			
**PKH ratio**	1,19	1,18			

**Table 3:** Comparison of blast proliferation by cell cycle analysis using PI labelling and PKH67 labelling (PKH fluorescence intensity (a.u.) at Day 0 / PKH fluorescence intensity (a.u.) at Day 4) in 7 AML patients stimulated or not by mixture of cytokines and incubated with a tetrapeptide AcSDKP (amide or not). Sample 1: PKH67 labelled cells cultured without the mixture of cytokines. Sample 2: PKH67 labelled cells cultured with the mixture of cytokines. Sample 3: cells with cytokines and AcSDKP-NH2 in 10^-9 ^M. Sample 4: cells incubated with AcSDKP in 10^-5 ^M to which cytokines were added after one day of culture. Sample 5: cells incubated with AcSDKP in 10^-9 ^M in which cytokines were added after one day of culture. Sample 6: cells incubated with AcSDKP-NH2 in 10^-9 ^M to which cytokines were added after one day of culture. Sample 7: cells without cytokines and AcSDKP-NH2 in 10^-9 ^M.

**Patients**	**Sample 1**	**Sample 2**	**Sample 3**	**Sample 4**	**Sample 5**	**Sample 6**	**Sample 7**
***Be***							
**PKH ratio**	1.24	1.25	1.26	1.27	1.28	1.3	1.26
**G0/1**	82	88	78	83	80	81	86
**S**	9	8	10	8	9	7	8
**G2+M**	9	4	12	9	11	12	6
***Dur***							
**PKH ratio**	1.2	1.5	1.51	1.49	1.48	1.51	1.25
**G0/1**	87	78	82	81	81	81	90
**S**	6	11	8	9	8	8	6
**G2+M**	7	11	10	10	11	11	4
***Ko***							
**PKH ratio**	1.51	1.49	1.50	1	1.12	1.11	1
**G0/1**	90	87	90	94	97	96	96
**S**	7	6	7	1	1	2	1
**G2+M**	3	7	3	5	2	2	3
**Patients**	**Sample 1**	**Sample 2**	**Sample 3**	**Sample 4**	**Sample 5**	**Sample 6**	**Sample 7**
***Pa***							
**PKH ratio**	1.18	2.01	2.1	1.8	1.75	1.75	1.13
**G0/1**	86	73	70	71	70	73	90
**S**	3	17	17	18	19	19	5
**G2+M**	11	10	13	11	11	8	5
***Ph***							
**PKH ratio**	1.18	1.5	1.5	1.31	1.32	1.33	1.11
**G0/1**	85	78	82	83	76	83	87
**S**	2	12	12	9	11	9	2
**G2+M**	13	10	6	8	13	8	11
***Al***							
**PKH ratio**	1	1.37	1.25	1.01	1.09	1.07	1
**G0/1**	95	83	89	93	94	91	92
**S**	1	8	5	1	1	1	1
**G2+M**	4	9	6	6	5	8	7
***Av***							
**PKH ratio**	1.1	1.6	2	1.1	1.09	1.02	1
**G0/1**	92	66	63	90	89	90	94
S	1	16	17	1	1	1	1
**G2+M**	7	18	20	9	10	9	5
